# Assessment of Stability of MIMU Probes to Skin-Marker-Based Anatomical Reference Frames During Locomotion Tasks: Effect of Different Locations on the Lower Limb

**DOI:** 10.3389/fbioe.2021.721900

**Published:** 2021-12-22

**Authors:** Giovanni Marco Scalera, Maurizio Ferrarin, Alberto Marzegan, Marco Rabuffetti

**Affiliations:** IRCCS Fondazione Don Carlo Gnocchi, Milano, Italy

**Keywords:** MIMU stability, gait, locomotor tasks, skin-mounted sensors, rehabilitation, wearable sensors

## Abstract

Soft tissue artefacts (STAs) undermine the validity of skin-mounted approaches to measure skeletal kinematics. Magneto-inertial measurement units (MIMU) gained popularity due to their low cost and ease of use. Although the reliability of different protocols for marker-based joint kinematics estimation has been widely reported, there are still no indications on where to place MIMU to minimize STA. This study aims to find the most stable positions for MIMU placement, among four positions on the thigh, four on the shank, and three on the foot. Stability was investigated by measuring MIMU movements against an anatomical reference frame, defined according to a standard marker-based approach. To this aim, markers were attached both on the case of each MIMU (technical frame) and on bony landmarks (anatomical frame). For each MIMU, the nine angles between each versor of the technical frame with each versor of the corresponding anatomical frame were computed. The maximum standard deviation of these angles was assumed as the instability index of MIMU-body coupling. Six healthy subjects were asked to perform barefoot gait, step negotiation, and sit-to-stand. Results showed that (1) in the thigh, the frontal position was the most stable in all tasks, especially in gait; (2) in the shank, the proximal position is the least stable, (3) lateral or medial calcaneus and foot dorsum positions showed equivalent stability performances. Further studies should be done before generalizing these conclusions to different motor tasks and MIMU-body fixation methods. The above results are of interest for both MIMU-based gait analysis and rehabilitation approaches using wearable sensors-based biofeedback.

## Introduction

Magnetic and inertial measurement units (MIMU) are widely adopted in human movement analysis due to their low cost, high miniaturization, power efficiency (Fong and Chan, 2010; Cuesta-Vargas et al., 2010; Tao et al., 2012), and their capacity to perform real-time analysis both in the laboratory and in real-world scenarios (Benson et al., 2018).

In the movement analysis field, joint angular kinematics represent key determinants for the discrimination between normal and pathological gait (Saunders et al., 1953; Perry, 1992) and for the clinical decision making process in rehabilitation (Ferrarin et al., 2015). Joint kinematics can be estimated by adopting either optical motion capture systems or wearable systems such as MIMU: in the former case, the position of reflective markers placed on bony landmarks is detected by stereophotogrammetric systems (Lencioni et al., 2019); in the latter MIMU measurements of acceleration, angular velocity and local magnetic field are fused together in order to obtain the estimation of the unit orientation (Sabatini, 2011; Bergamini et al., 2014; Caruso et al., 2020). Independently of the measurement system, a sufficiently rigid connection between the skeletal structure and the skin-attached markers or MIMU is assumed. Joint angles are then calculated as the relative orientation of two adjacent body segments, eventually through an intermediate anatomical calibration (Picerno et al., 2008; Cutti et al., 2010; Weygers et al., 2020).

However, although MIMU is becoming more and more widespread in motion tracking applications, users should be aware of the different sources of error that affect this methodology. Among them, sensor drift, magnetic interference, and, as with all methods based on skin-attached devices, the soft tissue artefacts are the most relevant in human motion applications (Hughes et al., 2021) and represent the major determinants in the orientation estimation errors (Cereatti et al., 2015).

In human motion tracking, the motion of the skin and the underlying soft tissue with respect to the bone is the source of the so-called soft tissue artefact (STA) (Cappozzo et al., 1996; Leardini et al., 2005). STA reduces the accuracy of actual bone motion measurement and is still an open issue in human movement reconstruction, with both marker-based systems (Camomilla et al., 2017) and wearable devices (Cereatti et al., 2017). STA depends on the physical characteristics of the subject, on the specific body segment, onto the precise site on which the marker/sensor is positioned (Cereatti et al., 2017; Camomilla et al., 2018) and on the performed motor task (Lafortune, 1991).

In an objective manner, STA was assessed (Peters et al., 2010) using markers mounted on pins inserted into bones (Cappozzo et al., 1996) or using imaging techniques such as fluoroscopy (Stagni et al., 2005). These procedures are invasive, expensive, and time-consuming. A complete description of the STA should include the amplitude and the direction of the marker position variation over time, the location of the marker, the body segment on which the marker is placed, the subject anthropometry, and the activity performed (Cereatti et al., 2017). These factors reveal the high specificity and variability of the phenomenon, thus suggesting the use of common metrics for results comparison between different studies (Cereatti et al., 2017). Modeling the STA affecting clusters of markers has unveiled two components: a rigid one and a non-rigid one (Dumas et al., 2014; Bonci et al., 2015), where only the STA rigid component actually affects the identification of a reference frame rigid with the segmented anatomy. While considering that MIMUs, as well as marker clusters mounted on a single rigid plate, substantially present only the STA rigid component, it is expected that the rigid component itself may suffer from artefacts due to soft tissues wobbling more than what has been observed in clusters of skin markers (Bonci et al., 2014). Therefore, in the case of a MIMU-based application, STA detrimental effect is particularly related to the unit weight/size and its attachment method (Forner-Cordero et al., 2008; Cleland et al., 2012) and to the unit possible repositioning (Decker et al., 2011).

Despite the large number of studies dealing with MIMU-based joint kinematics estimation, there are no indications in the literature on where MIMU should be positioned in order to minimize the error due to MIMU-body coupling. This study is focused on the assessment of MIMU mechanical stability, considered as an indirect measure of STA, during different locomotor tasks. The more the MIMU is unstable, the more STA will be relevant and will cause errors in joint angles calculations with respect to the ground truth. The discussion will provide hints on where MIMU should be positioned on lower limbs in order to minimize the effect of STA.

## Methods

### Experimental Approach and Stability Index

The stability of MIMU during three different locomotor tasks was investigated by measuring the MIMU oscillations with respect to the anatomical reference frame, defined according to a standard marker-based approach, considered as the gold standard. To track MIMU movements, on each considered anatomical segment, three not aligned hemispheric markers were attached on each MIMU case, defining an orthogonal technical reference frame (applying a Gram-Schmidt orthogonalization: a first axis passing through the two markers aligned with the longest MIMU edge; a second axis perpendicular to the plane defined by the three markers, and implicitly perpendicular to the previously defined axis; a third axis orthogonal to the two already defined ones) whose axes are approximately aligned with the axes of the anatomical reference frame. A 10-camera optoelectronic system (Smart Dx 700, BTS, Italy) was then used to capture both the technical and the anatomical reference frame. In particular, the movement of the technical reference frame of MIMU attached, respectively, to the thigh, the shank, and the foot was referred to as the movement of the anatomical reference frame of the correspondent body segment. The definition of the anatomical reference frame of each segment is fully reported in Rabuffetti et al. (2019). The stability of each MIMU was assessed calculating the standard deviations, during the execution of each motor task, of the angles between each versor of each frame according to the following equation:

$$\sigma_{ij}=std\left(\frac{180}{\pi }dot\left(v_{i},V_{j}\right)\right),$$

where the dot represents the dot product, v is the versor of the technical frame, V is the versor of the corresponding anatomical frame, *i* = x,y,z, and *j* = X,Y,Z.

The max of the nine standard deviations represents an Instability Index (InI), whose unit is degrees. In an ideal condition of total absence of instability, the above angles would have a constant value (correspondent to the fixed displacement between technical and anatomical reference frame), which would lead to a null standard deviation and InI = 0 deg.

Additionally, an alternative instability index was computed as the mean value of the nine standard deviation values in order to comparatively verify if instability occurs along preferred directions (in which case a large difference is expected between max and mean values) or it is evenly distributed among all MIMU spatial orientations (in which case max and mean values are expected to have the same order of magnitude). To quantify the differences between the magnitude orders, a linear regression analysis of Max and Mean Instability Indices was done, and the first order coefficient was extracted.

### Subjects and Tasks

Six adult subjects (18–53 age range, four males) were recruited and asked to perform at self-selected speed, barefoot overground gait, ascending and descending step negotiation, and sit-to-stand. Each task was repeated five times. The analysis was conducted considering the stance phase of each locomotor trial in order to minimize the calibration volume of the stereophotogrammetric system and to optimize markers’ detection and the whole transition for sit-to-stand. The anthropometric characteristics of participants are reported in Table 1. All participants gave informed consent to this study under the approval of the Local Ethics Committee.

**TABLE 1:** Details of the participants in the experimental sessions.

Gender	Age (years)	Height (m)	Mass (kg)	BMI (kg/m^2^)
M	53	1.77	75	23.9
F	26	1.79	69	21.5
M	18	1.76	63	20.3
M	31	1.69	64	22.4
F	27	1.68	57	20.2
M	32	1.68	55	19.5

### Skin Markers and MIMU Placement on the Body

**FIGURE 1:**
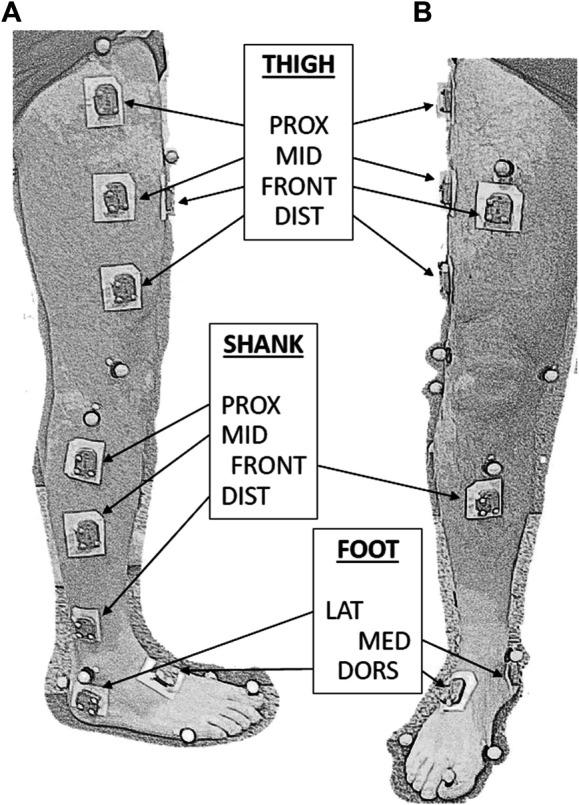
Sagittal **(A)** and frontal **(B)** views of a lower limb equipped with MIMUs (indicated by labels) and LAMB marker set. Three small-sized markers are attached to each MIMU to define the technical reference frame. MIMU are attached to the skin by a large bi-adhesive tape.

Retroreflective markers were positioned onto lower limbs specific bony landmarks, through stickers, according to the LAMB protocol (Rabuffetti et al., 2019). MIMU (WaveTrack, Cometa Systems srl, Italy) were attached with double-sided adhesive tape on eleven different positions of the dominant leg: four on the thigh, four on the shank, and three on the foot, as shown in Figure 1. The positions of MIMUs were determined in the following way: first, the lateral segment linking the great trochanter and the knee lateral epicondyle was identified for the thigh and the segment linking the knee lateral epicondyle and the lateral malleolus was identified for the shank. Then, the proximal, middle, and distal positions were identified at, respectively, 25, 50, and 75% of each lateral segment length. The frontal position of both thigh and shank was horizontally aligned to the middle position of the correspondent leg segment. On the foot, the lateral/medial positions were identified as the lateral/medial aspect of the calcaneus, under the correspondent malleolus, while the dorsal position was on the dorsal aspect of the middle foot.

The leg dominancy was determined by asking with which leg the participant kicks a ball. The MIMU longer dimension (size 35 × 24 × 10 mm, mass 10 g) was aligned with the longitudinal axis of each segment.

### Statistics

The considered overall dataset included a total of 1,320 Instability Indexes (6 subjects * four tasks * five trials * 11 MIMU positions). The statistical analysis was based on a non-parametric test (Kruskal-Wallis, *p* < 0.05), applied intra-segment, and a post hoc Tukey-Kramer multiple comparison test. All data processing and statistical analysis were performed in Matlab (The MathWorks Inc., United States).

## Results

The Instability Index InI ranged from 0.3° to 12.0°. No preferred anatomical direction characterized the Instability Index InI: the corresponding across-directions mean-based instability index values were approximately half (0.48, from a linear regression analysis) of the InI values.

**FIGURE 2:**
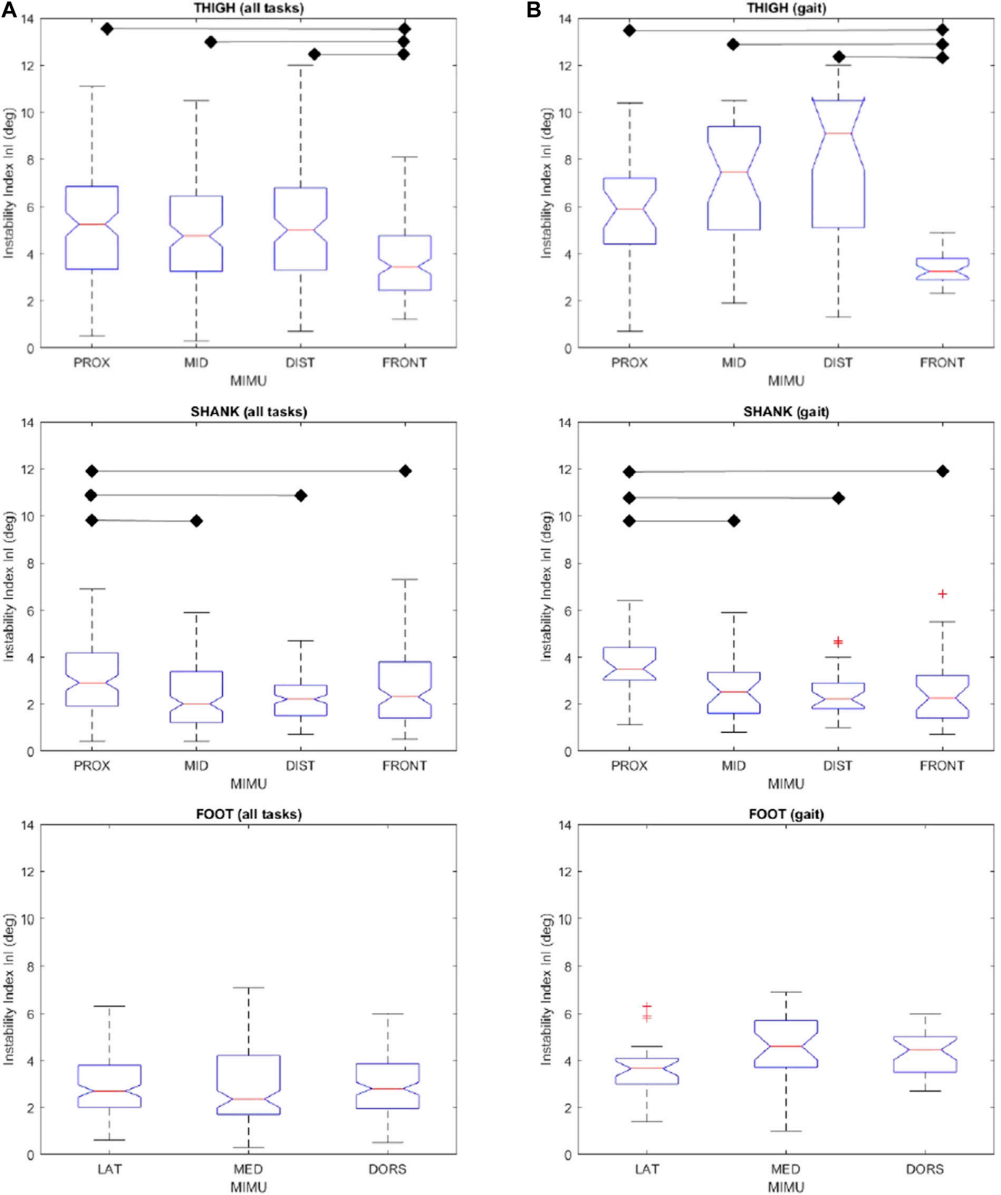
Instability index InI for the considered positions of the thigh, shank, and foot for all tasks **(A)** and for gait only **(B).** All values are expressed in degrees. Box and whiskers report median, quartile, and extreme values, “+” marks outlier values. Statistically different groups (Kruskal-Wallis *p* < 0.05) are indicated. Labels as in Figure 1.

As a summary of the results, Figure 2 shows InI values for all MIMU positions in each lower limb segment, considering both the whole dataset (N = 1,320) and only the gait dataset (N = 330). As a general picture, InI values were larger in the thigh (median value up to 9 deg) than in the shank and foot (up to less than 4 deg).

Kruskal-Wallis test revealed a significant difference (*p* < 0.05) among the considered positions for both thigh and shank. Conversely, differences between positions on the foot were not significant. Therefore, no further analysis was performed.

Multiple comparisons evidenced that the instability of thigh frontal position is significantly lower than that of all the thigh lateral positions. The overall higher stability of thigh frontal position is even more notable when considering only the gait task. Although InI ranking of thigh proximal, middle and distal lateral positions changed between the whole dataset and gait dataset, no statistically significant differences emerged among them in the two datasets.

All four positions on the shank reported comparable InI values with the exception of the shank lateral proximal position, whose values were significantly higher. These results were found both when considering all motor tasks and when considering the only gait.

For the interested readers, the dataset, which includes InI value (average value among the five repetitions) for each subject, motor task, and MIMU position, is available as a Supplementary file (RESULTS_InI_ALL_TASKS.xls).

## Discussion

The possibility of measuring the movement of human body segments is a key factor for the functional evaluation of locomotor disorders, the customization of rehabilitative approaches, the analysis of the efficacy of therapeutic treatments (surgical, pharmacological, physical etc.), and the choice of individualized orthotic/prosthetic solutions (Benedetti et al., 2017). The availability of wearable MIMU-based devices offers the opportunity to perform such evaluations with more affordable costs than those associated with laboratory-based stereophotogrammetric systems, provided that the measurements are sufficiently valid. Moreover, wearable devices open the possibility to provide real-time information about performed movements, useful to implement biofeedback-based rehabilitation of balance and gait (Carpinella et al., 2017).

This study is focused on the assessment of soft tissue artefacts that affect MIMU during common locomotor acts, with the aim of finding the most stable MIMU location on each segment of the lower limb. STA is an issue in the movement analysis field and occurs each time a skin-mounted marker or sensor is adopted. To isolate the effect of STA, avoiding other error contributes such as sensor drifts or magnetic disturbances, the adopted method relied only on optoelectronic measurements, treating MIMU as rigid bodies with a proper mass, shape, and dimensions and keeping them turned off during the whole experimental session.

The stability of each MIMU was assessed by the relative angular movement between the reference frame associated with the MIMU and the reference frame associated with the anatomical segments (identified by skin markers according to a consolidated gait analysis protocol (Rabuffetti et al., 2019)) where each probe is positioned. It is worth mentioning that also skin markers are prone to STA (Stagni et al., 2005). Therefore, the MIMU stability measured in the present study is not strictly relative to the underlying bones. However, skin markers negligible mass and the large inter-distances likely determine significantly smaller skin marker STA compared to MIMU associated STA. To verify this hypothesis, approaches based on bone-fixed transcutaneous pins or fluoroscopy, both unavailable at our facility, should be adopted. Anyway, it is reasonable to state that among different MIMU positions on a specific body segment, the larger are the MIMU movements wrt to the skin marker-based anatomical reference frame, the smaller is the stability of MIMU-segment coupling.

The use of the maximum value in the definition of the Instability Index was conservatively adopted to reflect the worst scenario. Still, it was verified that such instability did not exclusively or predominantly occur along a particular anatomical axis.

MIMU positions on the lower limb were selected according to criteria of reasonableness and widespread use, exploiting the lateral side of the limb and considering the medial side only in the case of the foot.

For gait and step negotiation trials, the analysis was conducted examining only the stance phase, thus considering the impulsive forces generated at foot-floor impact and the muscular, active contraction phase as the predominant cause of the STA.

As a general result, the MIMU instability on the thigh was higher than on the shank and foot: even in the worst positions of the shank and foot, InI values were lower or similar to the best ones in the thigh. This result highlights the different levels of criticality of MIMU positioning on the three segments of the lower limb: while on the thigh, the choice of MIMU location may determine significantly favorable/detrimental effects on STA, on the shank and foot, this choice is less critical.

Among the considered thigh positions, the frontal one, in the middle of the thigh length, obtained the lowest values of the InI index and so the highest stability. In order to reduce the STA effect during sit-to-stand, step negotiation, and gait analysis experiments, MIMU should be placed in this frontal position. Differences between proximal, middle, and distal placements of the lateral side of the thigh were not statistically significant.

**FIGURE 3:**
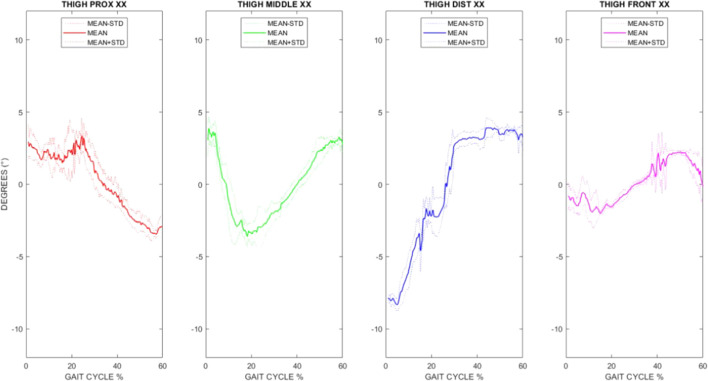
Time course of the angle between the longitudinal axis (X) of the thigh anatomical frame and the corresponding axis (x) of the proximal (red), middle (green), distal (blue), and frontal (magenta) technical frames for one representative subject. Mean curves (plus/minus one standard deviation) of five gait trials are reported, considering the stance phase.

The best choice of thigh frontal position is even more apparent when examining the average time course of the angle between the longitudinal axis (X) of the thigh anatomical frame and the longitudinal axis (X) of the technical frame during gait (as shown in Figure 3 for a representative subject). It is apparent the lower excursion of the angle obtained from the thigh frontal position. Similar trends were found in all subjects and selecting different angles among the nine.

While on the thigh, the results evidenced the existence of a most stable position to place the MIMU, from shank results, it is clear that the proximal one is the most unstable position. Although shank middle, distal, and tibial facets are seemingly equally valid choices, it may be convenient to put MIMU on the distal shank, considering its better performance in gait event detection and temporal parameters estimation (Pacini-Panebianco et al., 2018).

No differences emerged when MIMU were attached to the foot on the lateral and medial calcaneus and on the dorsal position. For practical considerations, the medial calcaneus position should be avoided to limit the possibility of scuffing the probes with the contralateral foot. In applications such as pedestrian tracking, MIMU is often positioned on the foot dorsum (Scapellato et al., 2005; Ferrari et al., 2016). However, in such free living applications, MIMU were fixed to shoelaces in order to reduce sensor jolt, which is in contrast to our laboratory analysis conducted in barefoot condition.

Although this study revealed which positions should be chosen/avoided in order to minimize STA, it is worth mentioning some specificities that limit possible generalizations of the results.

Among the factors that influence STA subjects, anthropometry has a primary role. Subjects included in this experimental campaign had a normal body mass index (BMI); it is plausible that in a cohort with different BMIs, such as an obese population or people with muscolo-skeletal abnormalities, the effect of the STA may be emphasized or show different behavior. In the sample of subjects here considered, individual results mostly—but not always—coincide with the average trends shown in Figure 2, meaning that in few specific cases, the most/least stable MIMU position could be different from those indicated by the group analysis. However, the limited number of subjects included in the present study does not allow us to extract information on how specific anthropometry may influence MIMU stability in the different body locations.

Another key factor in the STA assessment is the fixation method of the units. The present experiment was conducted using double-sided adhesive tape, which appears as a suitable choice for laboratory analysis of short duration. There are no indications on how the results hereby presented may vary with a different fixation system such as velcro elastic straps.

The results here presented do not include the swing phase of gait in order to minimize the calibration volume and optimize marker’s detection and reconstruction, a crucial aspect when angular displacement is computed from markers very close to each other, as those on the MIMU case considered for the present application. Therefore, STA generated during the swing phase are not considered in the present study, although it may be hypothesized that they should be less than those associated with the stance phase, at least as regards the wobbling components, since the maximum acceleration is expected at foot strikes, which are included in stance.

Lastly, the activities considered in this study are locomotor acts characterized by relatively low frequency components. More dynamic activities, such as sports applications where MIMU are actually widely adopted, may show different results and deserve further investigation. Other factors that could imply sport-specific results are the larger muscle contraction typically involved in sports activity and the increased muscle mass of athletes.

## Data Availability

The raw data supporting the conclusion of this article will be made available by the authors without undue reservation.
